# Filtering across Spatial Scales: Phylogeny, Biogeography and Community Structure in Bumble Bees

**DOI:** 10.1371/journal.pone.0060446

**Published:** 2013-03-27

**Authors:** Alexandra N. Harmon-Threatt, David D. Ackerly

**Affiliations:** 1 Department of Environmental Science, Policy and Management, University of California, Berkeley, California, United States of America; 2 Department of Integrative Biology, University of California, Berkeley, California, United States of America; 3 Department of Biology, Washington University in St. Louis, St. Louis, Missouri, United States of America; Field Museum of Natural History, United States of America

## Abstract

Despite the expansion of phylogenetic community analysis to understand community assembly, few studies have used these methods on mobile organisms and it has been suggested the local scales that are typically considered may be too small to represent the community as perceived by organisms with high mobility. Mobility is believed to allow species to mediate competitive interactions quickly and thus highly mobile species may appear randomly assembled in local communities. At larger scales, however, biogeographical processes could cause communities to be either phylogenetically clustered or even. Using phylogenetic community analysis we examined patterns of relatedness and trait similarity in communities of bumble bees (*Bombus)* across spatial scales comparing: local communities to regional pools, regional communities to continental pools and the continental community to a global species pool. Species composition and data on tongue lengths, a key foraging trait, were used to test patterns of relatedness and trait similarity across scales. Although expected to exhibit limiting similarity, local communities were clustered both phenotypically and phylogenetically. Larger spatial scales were also found to have more phylogenetic clustering but less trait clustering. While patterns of relatedness in mobile species have previously been suggested to exhibit less structure in local communities and to be less clustered than immobile species, we suggest that mobility may actually allow communities to have more similar species that can simply limit direct competition through mobility.

## Introduction

Understanding patterns of species diversity and assembly is a major objective of research in ecology, evolution and biogeography. The recent development of methods to integrate phylogenetics into community ecology–“phylogenetic community ecology”–makes it possible to simultaneously address spatial and temporal questions about how species assemble and what processes impact assemblage membership [1]. Recently, however, concern has been raised about the scales at which phylogenetic community methods are measured and whether expanding questions to biogeographical scales and considering a more diverse array of taxa could improve our understanding of community assembly [2]–[4].

Phylogenetic methods are commonly used to determine the phylogenetic clustering vs. evenness (i.e. the degree of relatedness), and the degree of phenotypic similarity or differentiation of community members in local communities, compared to null communities drawn from a larger, regional species pool [5]. Patterns of trait conservatism (i.e. the extent to which close relatives are phenotypically similar) provide a critical linkage between phylogenetic and phenotypic patterns, focusing on traits related to resource use and community structure. These methods can also be applied to greater spatial scales to help illuminate how patterns of phylogenetic relatedness change across scales and how biogeographical factors might also impact patterns seen at local and regional spatial scales. Of the studies that have considered possible effects of spatial scale on patterns of relatedness, most do not vary the size of the local assemblage but change the size of the regional species pool (e.g. [4]) which is known to influence statistical power [6]. Additionally, few studies consider both the importance of traits and relatedness in a single community (see [7], [8]).

Increasing the scale of analysis used for phylogenetic community analysis could also help expand studies to mobile taxa for which patterns are believed to arise at scales larger than those normally considered by community ecology (e.g., <1 km) [1]. High mobility can allow species to mediate competitive interactions quickly and may explain why some species appear randomly assembled at small spatial scales [2]. However, some mobile species such as hummingbirds were found to exhibit even trait dispersion in local communities [7], thus making the relationship between scale and mobility unclear. Despite concerns about the effect of spatial scale and mobility of organisms on local patterns of species diversity, the range of taxonomic systems addressed is still very low and most have limited mobility (e.g. plants, microbes or Collembola) over short time periods. Of the 24 papers reviewed by Vamosi et al. [9] for phylogenetic community structure, only 2 considered species that can move freely between local assemblages. Understanding patterns of phylogenetic community assembly for highly mobile organisms is particularly important as species with large home ranges are at higher risks for decline [10].

Bumble bees offer an excellent model group to test the impacts of spatial scale on patterns of community assembly. As generalist, large bodied pollinators, bumble bees (*Bombus spp.*) are likely to disproportionately provide pollination service to many crops and wildflowers [11] and are the primary pollinators in high altitude and high latitude environments [12]. Additionally, bumble bees are extremely strong fliers with recorded flight distances of up to 2.5 km for some species [13]. Thus, assessing patterns of community diversity of *Bombus* could provide both vital information for pollination service in sensitive areas and insight into local assemblage and biogeographical patterns of highly mobile species.

Using phylogenetic community methods we measured the trait conservatism of a focal trait and the phylogenetic and trait diversity of *Bombus* communities at local, regional and continental levels to assess patterns of species diversity across spatial scales. Tongue length was chosen as the focal trait because it has previously been linked to resource partitioning [14]–[16] and affects handling efficiency of flowers and nectar extraction [17]–[19]. Tongue length is also strongly correlated with wing length and other morphological characteristics that can affect foraging and competition [20], [21]. The importance of resource partitioning and community assembly based on tongue length, however, has also been called into question. In European communities, tongue length was found to be more similar than expected when compared to randomly created communities [22]–[24] which suggests that communities may be filtered by environmental factors and tongue length may be locally clustered (i.e. more similar among co-occurring species).

As a large, native eusocial bee, bumble bees are assumed to exhibit strong intra-generic competition, due to the high resource demand to support colonies. If closely related species or species with similar tongue lengths compete more strongly, we would expect communities to be evenly dispersed in terms of relatedness or trait distributions, respectively. Using the *Bombus* phylogeny [25] and information on communities, species pools, and tongue lengths, we were interested in 3 questions related to community assembly of *Bombus*: 1) Does tongue length show significant patterns of phylogenetic conservatism? 2) Are there non-random patterns of tongue length or relatedness among co-occurring *Bombus* species? 3) Do trait and phylogenetic patterns vary with spatial scale? As mobility may allow species to limit direct competition and assemble freely in local communities [2], we hypothesize that at local scales bumble bees will be neutrally assembled and that traits will also be neutrally assembled. We also propose that as spatial scales increase bumble bee communities will appear more phylogenetically clustered as the biogeographic processes, such as speciation in the New World, at larger spatial scales will increasingly filter communities.

## Methods

### Ethics Statement

Data on local assemblages were shared by researchers with the full knowledge that it would be used to analyze patterns of relatedness.

### Data collection

*Bombus* tongue length data were collected through literature searches in ISI Web of Science during the spring of 2009 using search terms: (Bombus or bumble*) and (proboscis or tongue). Additional sources were acquired by searching cited literature. Only data for the worker caste that was directly measured as the sum of prementum and glossa were used [26]. If multiple records existed for a bee species, the sample size weighted average of all records was used. All species in the sub-genus *Psythirus* were removed from analysis because they do not have a worker caste and their existence in a community is dependent on an appropriate host.

For clarity, local communities are referred to as assemblages [27] which are defined as species that co-occur spatially and temporally in a community and are potentially competing for and partitioning resources or other niche axes. Assemblages were identified by contacting authors and researchers with survey data on pollinators or *Bombus* to acquire original databases on bumble bee species presence in the Nearctic Ecozone. Original data were required because published data was typically pooled spatially or temporally. Only data that was collected from sites greater than one km apart and in which sampling was conducted across all plant species were used to ensure sites were distinct and no species were excluded by sampling a single plant species [28], [29]. All studies were designed to fully sample *Bombus* or pollinator diversity so although they varied in size and sampling intensity (Table 1) in all cases effort was made to fully capture diversity within the study. Additionally differences in sampling area and intensity were not correlated with species richness within or between studies. If sites were sampled repeatedly, only the sampling date with the highest diversity, a proxy for most complete sampling of the site, was chosen. Abundance data were excluded from the analysis because they were not available for all sites.

**Table 1:** Information on local assemblages included in analysis including name of contributor, number of sites provided, location and size of area sampled, duration of sampling, *Bombus* richness in sites, regional species pool as determined using Williams(1996) equal area grid cells and publication information.

Data contributor	Number of sites	Location	Site Size (ha)	Sampling time	Richness	Regional Species Pool	Publication
S. Colla	1	Southern Ontario	0.4	full transect walked	6	18	Colla and Packer(2008)
E. Evans	2	Minnesota	0.06	60–120 mins	5–7	13	unpublished
R. Hatfeld	20	California	0.377	45 mins	2–8	17	Hatfield and Lebuhn (2007)
S.Hendrix	13	Iowa	.5–1	60–120 mins	2–6	15	Hendrix, Kwaiser and Heard (2010); Kwaiser and Hendrix (2008); unpublished
C. Kearns	15	Colorado	0.385	variable	2–4	20	Kearns and Oliveras(2009)
R. Malfi and N. Williams	10	Pennsylvania and New Jersey	0.5	120 mins	4–6	16	unpublished
A. Tripodi	49	Tennessee and Arkansas	.02–5	15–60 mins	2–5	11,13	unpublished

To test for non-random patterns in observed communities, regional species pools were used to generate null communities for comparison with observed community phylogenetic distance and trait distributions. Regional species pools were defined based on equal area grid cells, following Williams [30]. Each grid cell covers approximately 611,000 km^2^ of the earth's surface (∼6.2 degrees latitude and 10 degrees longitude on average). Using DiscoverLife.org, a freely available database that pools occurrence and location records from museums and databases of global species occurrence, we determined the species that occurred in each grid cell within Nearctic regions of North America (hereafter Nearctic) and compared these to published records of species occurrence when possible. Only data points that had been verified by a taxonomist and georeferenced were used from the Discover Life database. Using predefined regional areas helps limit the variability in species pool size and definition across studies, which can significantly impact power of analysis [31].

To determine if species trait and phylogenetic structure appear at spatial scales larger than local communities, regional species pools were then compared to the entire Nearctic (continental) pool and the continental pool was compared to the global species pool.

## Analysis

All analyses were conducted in **R** 2.10.1 using the *picante* package [32] with scripts written by the first author.

### Trait Conservatism

To determine levels of trait conservatism, we calculated Blomberg's K value, a metric for describing the distribution of phenotypic variation across the tips of a phylogeny [33]. A value of K = 1 is expected for a trait whose distribution matches the expectations for simple random-walk Brownian motion evolution across a phylogeny. A value of K>1 suggests higher trait conservatism while K<1 shows lower trait conservatism (relative to a random-walk model). A tip-swap null model can be used to test for the presence of phylogenetic signal by comparing the observed K-value to a null distribution based on randomizing trait values across the tips of the tree (N = 999). The expected K value under this null distribution is very low (<0.2), so significant conservatism can be detected even for K values that are much lower than those expected under Brownian motion [34].

We used the ultrametric, gap-coded, phylogenetic tree published by Hines [25], which is a time calibrated version of the Cameron et al. [35] tree. Species for which we did not have tongue length data were removed from the phylogeny. We analyzed trait conservatism for all species with published trait data (n = 79) and those that occur in Nearctic areas (n = 34) separately to determine if there were differing patterns of trait conservatism in the Nearctic areas, which were shown to be a more recent introduction and showed more rapid diversification than in the Old World [25].

### Phylogenetic Assemblage Analysis

Phylogenetic community analysis can identify patterns of relatedness in assemblages compared to null assemblages via several metrics. Here we use: 1) Mean Nearest Neighbor Distance (MNND) and 2) Mean Phylogenetic Distance (MPD) as defined and implemented in *picante* [32]. Using the phylogenetic tree, a pairwise phylogenetic distance matrix was created based on the branch lengths separating each pair of species. MNND calculates the phylogenetic distance between a species and the nearest related neighbor in an assemblage and provides analysis of phylogenetic clustering of closest relatives. MPD, in contrast, calculates the mean phylogenetic distance separating all assemblage members from each other and allows us to analyze the overall relatedness of the assemblage members.

For comparison, mean null values of MNND and MPD are calculated from 999 randomly generated assemblages with species richness equal to each of the observed assemblages and species selected at random from the regional species pool of the observed community ('taxa labels' null model in *picante*). The observed value is then ranked compared to the null values and the p-value is the rank/1000. From MNND and MPD corresponding z- score standardizations referred to as the Nearest Taxon Index (NTI) and Net Relatedness Index (NRI) using the mean and standard deviation are typically calculated to allow comparison across groups. Positive values of NTI and NRI indicate clustering of species in an assemblage compared to the nulls. Multiplying by negative one allows the indices to have more intuitive meaning with negative values indicating phylogenetic evenness and positive values indicating clustering. However, as highlighted by Cooper et al. [36], the null distribution of MPD is skewed, creating a bias towards negative NRI values (also see [37]). Thus, to determine trends across assemblages we use a Wilcoxon test to compare the p-values of the observed MNND/MPD to the expected median of the distribution for p-values, which is 0.5. Although NTI is not expected to have this bias, for consistency, we used the same Wilcoxon test on the MNND values. Using the W+ value calculated by the Wilcoxon test, we calculated Z scores and true p-values. To be consistent with the meaning of NRI and NTI, all Z-scores reported are multiplied by -1 with negative values suggesting evenness and positive values suggesting clustering. For all analyses, the species pool is limited to species for which we have trait data. This allows comparison between trait and phylogenetic data and was not found to significantly affect the results when Z-values were compared using a Paired Wilcoxon Signed Rank Test (p = 0.677).

### Trait-based Analysis

MNND and MPD are conventionally used to describe the phylogenetic distance between assemblage members. Here we use similar metrics to test for filtering of traits in assemblages called: Mean Nearest Trait Distance (MNTD) and Mean Trait Distance (MTD). MNTD and MTD are equivalent to MNND and MPD, respectively. Using the tongue length data for Nearctic species we created a phenotypic distance matrix of all species and calculated MNTD and MTD using the same method as above. Just as with phylogenetic distance, observed scores that are larger than nulls indicate limiting trait similarity in an assemblage. Positive Z-scores of MNTD and MTD indicate trait filtering.

To determine if tongue length was consistently spaced along a trait axis, potentially limiting competition within a site, we calculated the standard deviation of the successive neighbor distance when divided by the trait range within the assemblage (SDNN_r_) [38]–[40]. For each observed assemblage we sorted the tongue lengths of members from lowest to highest and then calculated the range of tongue lengths, the difference between successive tongue length values and the standard deviation of these differences. This standard deviation was divided by the range of tongue lengths in the assemblage. Assemblages with less than 3 species were removed because it is impossible to obtain a non-random spacing pattern for 2 species. We then tested whether the traits found in an assemblage had more even spacing of the trait (SDNNr) compared to 999 null assemblages of the same size drawn from the regional pool. We also tested whether the trait range in observed communities was smaller than the null assemblages which would suggest environmental filtering. P-values of trait metrics were compared using the Wilcoxon test as described above.

### Regional Pool Analysis

To assess the patterns of trait similarity and phylogenetic relatedness within the Regional Pool (Nearctic areas), we calculated the MNND, MPD, MNTD, MTD, SDNNr and range using trait and phylogenetic distance matrices for each of the 45 regional assemblages, using the entire Nearctic as the species pool for comparison.

### Continental Pool Analysis

We also calculated MNND, MPD, MNTD, MTD, SDNNr and range for the Nearctic assemblage compared to the global species pool to compare patterns of phylogenetic and trait distance within the continent compared to the global species pool. We were able to use the Z-score and p-value of the observed community compared to the null distribution because only one assemblage (the Nearctic) was examined.

## Results

### Trait Conservatism

**Figure 1:**
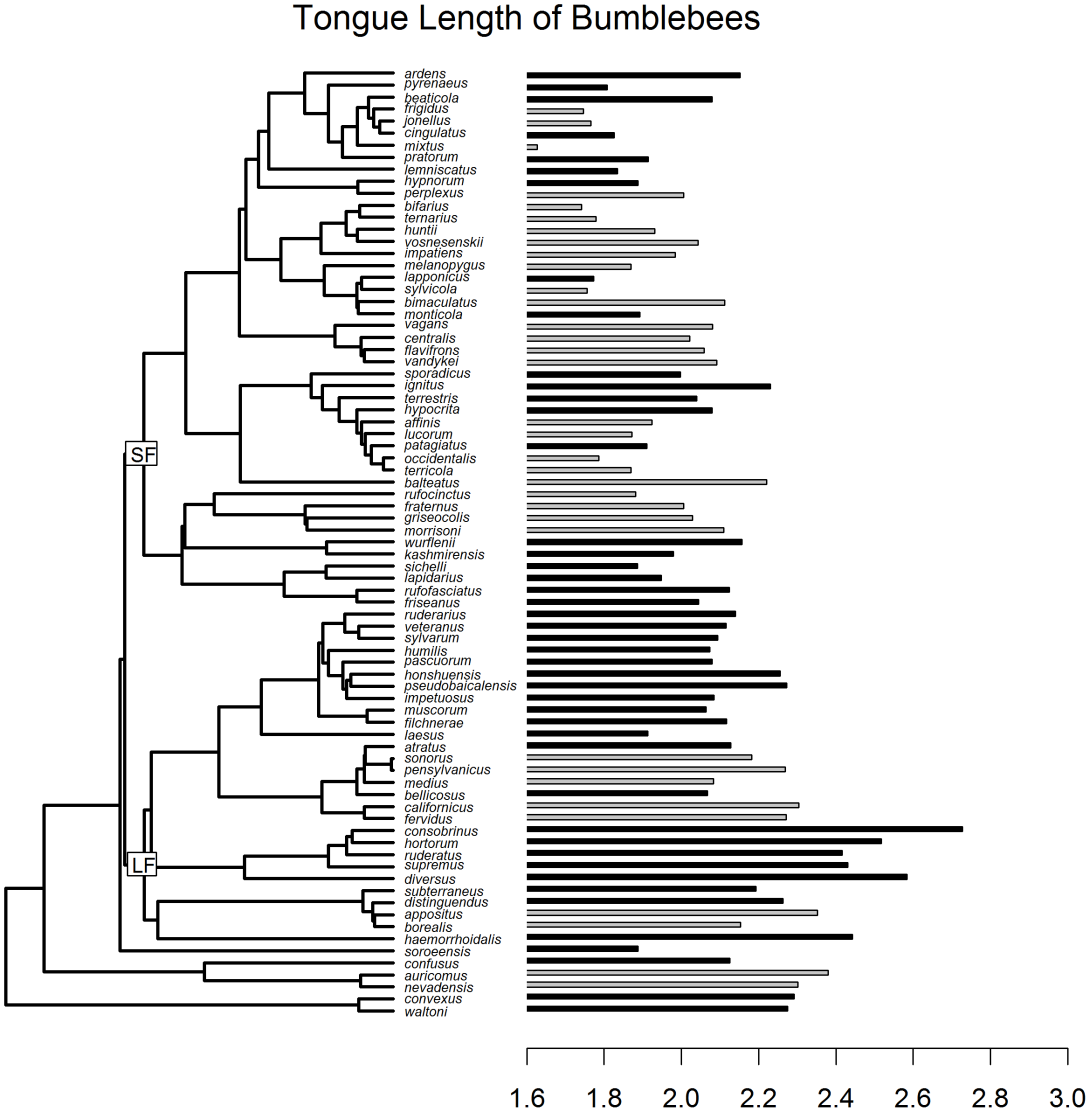
Bi-plot of the phylogeny of species with trait values (n = 79) and the associated tongue length measured in ln(mm). Grey bars indicate species found in the Nearctic. Short faced (SF) and long faced (LF) sister clades are labeled to demonstrate the association with tongue length. Taxa labels are available in the Supplementary Table 1 with trait values.

We found a total of 18 published studies and 1 unpublished Master's thesis with measured worker tongue length spanning 79 species globally and 34 species in the Nearctic (see Table S1, **Figure 1**) to analyze trait conservatism across the phylogeny. For the global sample, Blomberg's K for tongue length was 0.717 while for Nearctic species K = 0.719 which is similar to other morphological traits for animals [33]. Both global and Nearctic areas showed highly significant phylogenetic signal of the trait on the phylogeny, relative to the tip swap null (p<0.001).

### Phylogenetic Assemblage Analysis

**Figure 2:**
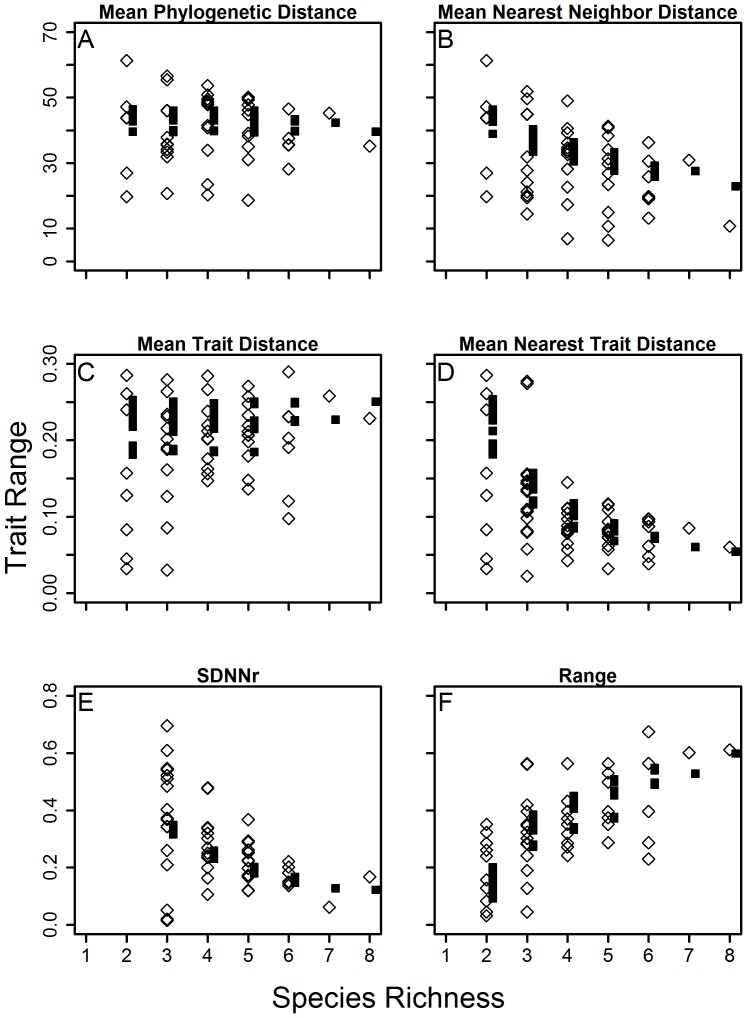
Plot of observed values (diamonds) in communities and expected values (squares) vs. richness for a) Mean Phylogenetic Distance, b) Mean Nearest Neighbor Distance, c) Mean Trait Distance, d) Mean Nearest Trait Distance, e) SDNNr and f) Range.

We identified 110 assemblages in 8 of the 45 grid cells in Nearctic areas to analyze tongue length and relatedness across co-occurring species. Richness ranged from 2 to 8 species across assemblages and regional species pools for these assemblages ranged from 11 to 20 species (see Table 1). When tested for clustering of phylogenetic distance and nearest neighbor distance, local assemblages were significantly clustered for MPD (Wilcoxon signed-rank test of MPD: Z = 2.159, p = 0.031, **Table 2**, **Figure 2**).

**Table 2:** Z scores and p-values of relatedness and tongue length for various scales and measures of similarity.

		Local		Regional		Continental	
	Metric	Z	p	Z	p	Z	p
**Relatedness**	MNND	1.737	0.082	2.477	0.013	4.387	0.001
	MPD	2.159	0.031	1.433	0.152	3.490	0.001
**Trait**	MNTD	3.887	0.000	0.051	0.959	1.254	0.119
	MTD	4.933	0.000	−1.134	0.257	0.215	0.416
	range	3.754	0.000	0.386	0.699	1.267	0.122
	SDNNr	0.952	0.341	2.590	0.009	0.748	0.227

Local assemblages (n = 110) represent co-occurring species and the species pool is the regional gridcell the assemblage is within. Regional assemblages (n = 45) are the species in each grid cell compared to a species pool of all Nearctic Species. The continental assemblage (n = 1) consists of all Nearctic species compared to all *Bombus* globally.

### Trait Assemblage Analysis

For the same 110 observed communities above, the trait analysis revealed that tongue length had significantly lower nearest trait distance (MNTD) and significantly more similar overall tongue lengths (MTD) in observed assemblages compared to nulls (Wilcoxon signed-rank test, MNTD: Z = 3.887, p>0.001 and MTD: Z = 4.933, p>0.001, respectively, **Table 2**, **Figure 2**). Additionally, tongue length was not evenly spaced in assemblages (SDNNr: Z = 0.952, p = 0.5341) and only five of 84 assemblages were more evenly spaced than nulls. The range of tongue lengths was significantly lower than expected by chance, indicating trait clustering in local communities (Z = 3.754, p<0.001)

### Regional Pool Analysis

Phylogenentic metrics and trait metrics were calculated at the regional and continental level to determine if increasing scale of analysis altered patterns of dispersion for bumble bee assemblages. Regional species pools contained species with lower nearest neighbor phylogenetic distances than the nulls drawn from the continental pool (Wilcoxon signed-rank test of MNND: Z = 2.477, p = 0.013, Table 2). Regional pools were not significantly different from nulls for MPD, MNTD, MTD or range. However, SDNNr was smaller in regional assemblages compared to the species pool suggesting evenness for the regional assemblages (Z = 2.59, p = 0.009).

### Continental Pool Analysis

When compared to the global species or trait pools, the Nearctic had highly significant phylogenetic relatedness for both MNND and MPD (MNND: Z = 4.387, p = 0.001, MPD: Z = 3.490, p = 0.001) but was not significant for any trait measure.

### Filtering across spatial scales

**Figure 3:**
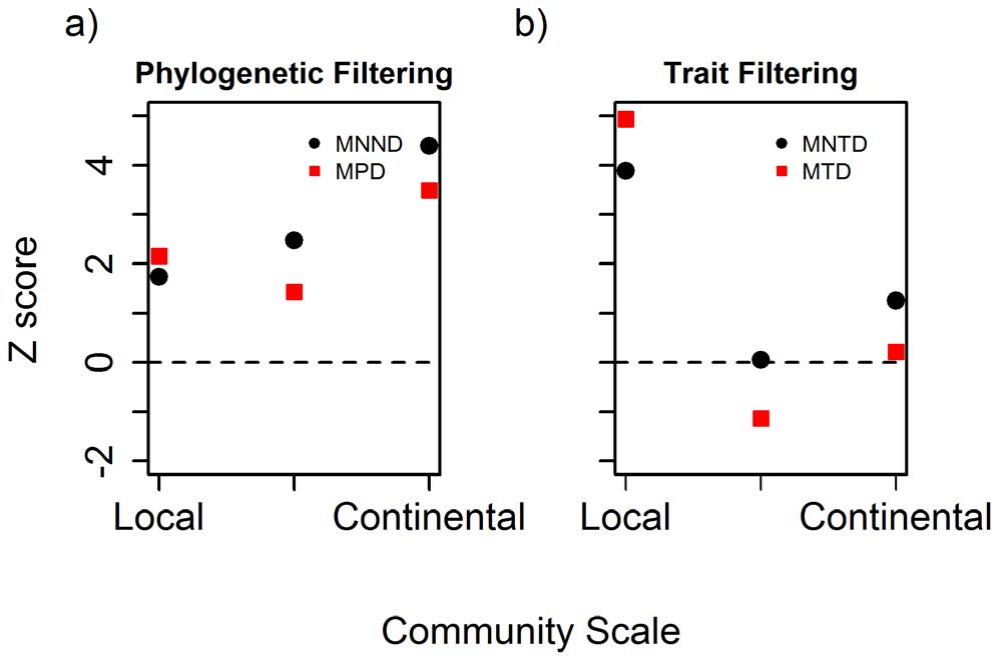
Z-scores of observed communities when compared to randomly generated null communities at various spatial scales for: a) Mean Nearest Neighbor Distance (MNND) and Mean Pairwise Distance (MPD) and b) Mean Nearest Trait Distance (MNTD) and Mean Trait Distance (MTD) for trait similarity. The dotted line represents the null expectation if communities are randomly organized. Positive values suggest clustering while negative values suggest evenness.

Using the results above we can look at trends across the 3 spatial scales by plotting the Z-scores compared to null communities. Results for MNND reveal increasing phylogenetic clustering across spatial scales but metrics were similar at local and regional scales (see **Figure 3**a.). Trait analysis had the most clustering at local spatial scales and the least at regional scales (see **Figure 3**b.).

## Discussion

Under the competition-relatedness hypothesis [41], species that are closely related are expected to compete more strongly if traits mediating competition are highly conserved, thus causing local communities to be evenly dispersed to limit trait and phylogenetic similarity. Alternatively, for species that are highly mobile and can mediate competition quickly we might expect local communities to be randomly assembled. We found that local assemblages of *Bombus* had significant clustering of mean phylogenetic distance (MPD) but not of mean nearest neighbor distance which suggests that local assemblages are overall closely related but not simply made up of sister taxa. Traits in local assemblages were also clustered for mean nearest trait distance (MNTD), mean trait distance (MTD) and range which suggests that local assemblages have more similar tongue lengths than expected by chance. As tongue length has strong phylogenetic signal and is prone to convergence on both the global and Nearctic phylogeny, the trait clustering is consistent with phylogenetic clustering. This pattern arises despite high levels of variability in worker size in *Bombus* nests [42], [43] and evidence that alternative methods for measuring may introduce additional error to the analysis [44]. The lack of even trait spacing (SDNNr) also constitutes a lack of evidence for niche partitioning in these local communities.

Similarity in trait values and higher than expected relatedness among co-occurring species may suggest that other biotic and abiotic features are more important in structuring local *Bombus* communities. Competitive interactions, depending on their strength, can cause assemblages to be clustered or even [45]. In hummingbirds, strong competitive interactions for floral resources cause beak length, which effects flower foraging, to be evenly dispersed [7]. We observed the opposite pattern for bumble bees which may suggest that competition for floral resources does not cause exclusion. When competition does not play a significant role, pollinators have been found to share closely related floral resources [46] and floral communities may favor similarities in pollinator foraging traits among co-occurring taxa in a location [47]. If local floral communities are strongly clustered phenotypically, and traits related to foraging are phylogenetically conserved, one would expect the pollinator communities to be more phylogenetically clustered as well [24], [47], [48], as observed here. We suggest that future work look into the similarity between morphological traits of bees and floral characteristics in local communities. Alternatively, phylogenetic clustering could arise if other resources are limiting and the associated morphological trait is phylogenetically conserved. Bumble bees share similar nesting characteristics (e.g. pocket-makers or pollen storers) [49] and thus nesting sites and materials could be limiting [50], [51].

At regional and continental scales no significant pattern was found for trait metrics but MNND was clustered at both scales and MPD was clustered continentally. The lack of significant clustering at the regional scale is supported by significant SDNNr suggesting even spacing of traits. Clustering of MNND and MPD at the continental scale may be a reflection of geographic barriers to bumble bees reaching the New World while the significant clustering of MNND and lack of clustering of trait metrics in regional areas may suggest radiation to fill the various niches. These regional radiations may also explain why tongue length is less conserved along the phylogeny than expected under Brownian motion. So although it has been suggested that assemblages of mobile species should have less signal of phylogeny over large spatial scales [1] than immobile species, we contend that similar patterns for mobile and immobile organisms may be observed with the expansion of spatial scales, but this deserves further testing. The increasing degree of clustering observed with scale suggests that environmental filtering may be significant across scales but with potentially different forces at work, such as local floral resources influencing assembly processes, regional radiations, dispersal to the New World, etc. It is interesting to note that only MTD at the regional level detects evenness, suggesting that despite increasing evenness of the trait across scales it is still clustered overall. We suggest these patterns be explored at spatial scales that are relevant for highly mobile organisms and this may require a bridging of biogeographical and community ecology methods.

Recent studies in the United Kingdom reported that the observed declines in bumble bee diversity disproportionately affect longer tongued species compared to co-occurring shorter tongued species [52]. This pattern along with the significant conservatism of this trait suggests that some clades are at a higher risk of declines than others. Additionally, the relatedness of assemblages observed would suggest that some communities may be at high risk of coextinctions of closely related species, as found by Rezende et al. [46]. As a result some communities and subgenera of bees may need additional consideration for conservation efforts.

## Supporting Information

Table S1 **Tongue lengths of all Bombus species recorded during study and source.** Note: A weighted average was used for species with multiple published measurements. Species are in the same order as in Figure 1 except Psythirus (shaded grey) which were excluded. Subgeneric classification is based on Williams et al. (2008). *indicates those in Nearctic region
(DOCX)
