# Supplementary material for: Filtering across Spatial Scales: Phylogeny, Biogeography and Community Structure in Bumble Bees
Source: PLoS One. 2013 Mar 27;8(3):e60446. doi: 10.1371/journal.pone.0060446 (PMC3609857; doi:10.1371/journal.pone.0060446)
Supplement: Table S1 — Tongue lengths of all Bombus species recorded during study and source. Note: A weighted average was used for species with multiple published measurements. Species are in the same order as in Figure 1 except Psythirus (shaded grey) which were excluded. Subgeneric classification is based on Williams et al. (2008). *indicates those in Nearctic region (DOCX) [file pone.0060446.s001.docx]

| species | subgenus | ln(tongue length) | Author(s) |
| --- | --- | --- | --- |
| *ardens* | Pyrobombus | 2.152 | Inoue and Yokoyama (2006) |
| *pyrenaeus* | Pyrobombus | 1.808 | Goulson et al. (2008) |
| *beaticola* | Pyrobombus | 2.079 | Inoue and Yokoyama (2006) |
| *frigidus** | Pyrobombus | 1.746 | Macior (1974) |
| *jonellus** | Pyrobombus | 1.766 | Goulson et al (2005), Ranta (1983), Goulson et al. (2008) |
| *cingulatus* | Pyrobombus | 1.826 | Pekkarinen (1979) |
| *mixtus** | Pyrobombus | 1.627 | Macior (1974) |
| *pratorum* | Pyrobombus | 1.914 | Goulson et al. (2005); Ranta (1983); Goulson et al.(2008) |
| *lemniscatus* | Pyrobombus | 1.834 | Williams et al.(2009) |
| *hypnorum* | Pyrobombus | 1.887 | Ranta (1983); Pekkarinen (1979); Goulson et al. (2008) |
| *perplexus** | Pyrobombus | 2.006 | Medler (1962) |
| *bifarius** | Pyrobombus | 1.741 | Macior (1974); Medler (1962); Bowers (1985) |
| *ternarius** | Pyrobombus | 1.779 | Medler (1962); Harder (1983) |
| *huntii** | Pyrobombus | 1.931 | Medler (1962); Bowers (1985) |
| *vosnesenskii** | Pyrobombus | 2.043 | Medler (1962) |
| *impatiens** | Pyrobombus | 1.984 | Medler(1962) |
| *melanopygus** | Pyrobombus | 1.87 | Macior (1974) |
| *lapponicus* | Pyrobombus | 1.773 | Pekkarinen (1979) |
| *sylvicola** | Pyrobombus | 1.756 | Macior (1974) |
| *bimaculatus** | Pyrobombus | 2.112 | Medler(1962); Harder (1983) |
| *monticola* | Pyrobombus | 1.892 | Goulson et al (2005); Pekkarinen (1979) |
| *vagans** | Pyrobombus | 2.081 | Medler(1962) |
| *centralis** | Pyrobombus | 2.022 | Macior (1974); Bowers (1985) |
| *flavifrons** | Pyrobombus | 2.059 | Inouye (1980); Macior (1974); Bowers (1985) |
| *vandykei** | Pyrobombus | 2.092 | Brooks MS thesis (1999) |
| *sporadicus* | Bombus | 1.997 | Pekkarinen (1979) |
| *ignitus* | Bombus | 2.23 | Inoue and Yokoyama (2006) |
| *terrestris* | Bombus | 2.04 | Goulson et al. (2005); Corbet et al. (1995); Pekkarinen (1979); Goulson et al.(2008) |
| *hypocrita* | Bombus | 2.079 | Inoue and Yokoyama (2006) |
| *affinis** | Bombus | 1.924 | Medler (1962) |
| *lucorum** | Bombus | 1.872 | Goulson et al. (2005); Pekkarinen (1979); Medler (1962); Goulson et al.(2008), Ranta (1983) |
| *patagiatus* | Bombus | 1.91 | Williams et al. (2009) |
| *occidentalis** | Bombus | 1.786 | Macior (1974); Medler (1962); Bowers (1985) |
| *terricola** | Bombus | 1.869 | Medler (1962) |
| *balteatus** | Alpinobombus | 2.221 | Medler (1962); Macior (1974) |
| *rufocinctus** | Cullumanobombus | 1.882 | Medler (1962); Bowers (1985) |
| *fraternus** | Cullumanobombus | 2.006 | Medler (1962) |
| *griseocollis** | Cullumanobombus | 2.029 | Medler (1962) |
| *morrisoni** | Cullumanobombus | 2.11 | Medler (1962) |
| *wurflenii* | Alpigenobombus | 2.157 | Medler (1962); Goulson et al. (2008) |
| *kashmirensis* | Alpigenobombus | 1.979 | Williams et al.(2009) |
| *sichelli* | Melanobombus | 1.886 | Williams et al.(2009) |
| *lapidarius* | Melanobombus | 1.948 | Goulson et al (2005); Ranta (1983); Corbet et al (1995); Goulson et al. (2008) |
| *rufofasciatus* | Melanobombus | 2.124 | Williams et al.(2009) |
| *friseanus* | Melanobombus | 2.045 | Williams et al.(2009) |
| *ruderarius* | Thoracobombus | 2.14 | Goulson et al (2005) |
| *veteranus* | Thoracobombus | 2.116 | Ranta (1983); Goulson et al. (2008) |
| *sylvarum* | Thoracobombus | 2.094 | Goulson et al.(2005); Medler (1962); Goulson et al.(2008) |
| *humilis* | Thoracobombus | 2.073 | Medler (1962); Goulson (2008) |
| *pascuorum* | Thoracobombus | 2.079 | Goulson et al (2005); Ranta (1983); Corbet et al (1995); Pekkarinen (1979); Goulson et al. (2008) |
| *honshuensis* | Thoracobombus | 2.256 | Inoue and Yokoyama (2006); Suzuki (2007) |
| *pseudobaicalensis* | Thoracobombus | 2.272 | Ishii et al (2008) |
| *impetuosus* | Thoracobombus | 2.084 | Williams et al.(2009) |
| *muscorum* | Thoracobombus | 2.064 | Goulson et al (2005); Pekkarinen (1979) |
| *filchnerae* | Thoracobombus | 2.117 | Williams et al.(2009) |
| *laesus* | Thoracobombus | 1.913 | Williams et al.(2009) |
| *atratus* | Thoracobombus | 2.128 | Arbulo et al.(2011) |
| *sonorus** | Thoracobombus | 2.182 | Medler (1962) |
| *pensylvanicus** | Thoracobombus | 2.269 | Medler (1962) |
| *medius** | Thoracobombus | 2.083 | Medler (1962) |
| *bellicosus* | Thoracobombus | 2.067 | Arbulo et al.(2011) |
| *californicus** | Thoracobombus | 2.304 | Macior (1974) |
| *fervidus** | Thoracobombus | 2.271 | Medler (1962) |
| *consobrinus* | Megabombus | 2.728 | Pekkarinen (1979); Suzuki et al.(2007) |
| *sylvestris* | Psythirus | 1.887 | Goulson (2008) |
| *bohemicus* | Psythirus | 1.946 | Goulson (2008) |
| *rupestris* | Psythirus | 1.946 | Goulson (2008) |
| *campestris* | Psythirus | 1.932 | Goulson (2008) |
| *insularis** | Psythirus | 2.102 | Macior (1974) |
| *hortorum* | Megabombus | 2.518 | Goulson et al (2005); Ranta (1983); Corbet et al (1995); Pekkarinen (1979); Goulson et al. (2008) |
| *ruderatus* | Megabombus | 2.416 | Goulson et al (2005); Medler (1962); Goulson et al. (2008) |
| *supremus* | Megabombus | 2.43 | Williams et al.(2009) |
| *diversus* | Megabombus | 2.584 | Inoue and Yokoyama (2006); Suzuki (2007) |
| *subterraneus* | Subterraneobombus | 2.193 | Goulson et al (2005); Medler (1962) |
| *distinguendus* | Subterraneobombus | 2.263 | Goulson et al (2005); Medler (1962); Goulson et al.(2008) |
| *appositus** | Subterraneobombus | 2.352 | Macior (1974); Medler (1962) |
| *borealis** | Subterraneobombus | 2.153 | Grixti et al (2009) |
| *haemorrhoidalis* | Orientalibombus | 2.443 | Dayal and Rana(2007) |
| *soroeensis* | Kallobombus | 1.887 | Goulson et al (2005); Medler (1962); Goulson et al. (2008) |
| *confusus* | Bombias | 2.125 | Medler (1962) |
| *auricomus** | Bombias | 2.38 | Medler (1962) |
| *nevadensis** | Bombias | 2.301 | Macior (1974); Medler (1962) |
| *convexus* | Mendacibombus | 2.292 | Williams et al.(2009) |
| *waltoni* | Mendacibombus | 2.275 | Williams et al.(2009) |

Table S1.
